# Physicochemical analyses of a bioactive 4-aminoantipyrine analogue - synthesis, crystal structure, solid state interactions, antibacterial, conformational and docking studies

**DOI:** 10.17179/excli2016-477

**Published:** 2016-10-26

**Authors:** Mohammad Sayed Alam, Dong-Ung Lee

**Affiliations:** 1 Division of Bioscience, Dongguk University, Gyeongju 780-714, Republic of Korea; 2 Department of Chemistry, Jagannath University, Dhaka 1100, Bangladesh

**Keywords:** 4-aminoantipyrine, crystal structure, molecular interactions, antibacterial, docking

## Abstract

A novel Schiff base derivative of 4-aminoantipyrine, that is, (*E*)-4-(2-methoxybenzylideneamino)-1,5-dimethyl-2-phenyl-1*H*-pyrazol-3(2*H*)-one (MBA-dMPP), was synthesized and characterized by FT-IR, ^1^H-NMR, and EI-MS. Single-crystal X-ray diffraction data revealed MBA-dMPP adopts a *trans* configuration around its central C=N double bond, and forms orthorhombic crystals. XRD revealed that MBA-dMPP possess two different planes, in which the pyrazolone and benzylidene groups attached to C9 of the pyrazolone ring are almost coplanar and the phenyl ring connected to the N1 atom of the pyrazolone moiety lies in another plane. The intermolecular, host-guest C-H…O, C-H…N, and C-H…C van der Waals interactions were found to form a 3D network and confer stability to the MBA-dMPP crystal structure. The quantitative and qualitative solid state behaviors of MBA-dMPP were subjected to 3D Hirshfeld surface analysis and 2D fingerprint plotting. Reciprocal H…H contacts contributed most (52.9 %) to the Hirshfeld surface, followed by C…H/H…C contacts (30.2 %), whereas, O…H/H…O and N…H/H…N interactions contributed 15.5 % to the Hirshfeld surface. Electrostatic potentials were mapped over the Hirshfeld surface to analyze electrostatic complementarities within the MBA-dMPP crystal. In addition, geometrical descriptors were also analyzed to the extent of surface interactions. MBA-dMPP was also investigated for *in vitro* antibacterial activity against Gram-positive and Gram-negative bacterial strains, and showed highest activity against *Bacillus cereus *(MIC = 12.5 μg mL^-1^) and *Salmonella* *tythimurium* (MIC = 50 μg mL^-1^). *In silico* screening was conducted by docking MBA-dMPP on the active site of S12 bacterial protein (an important therapeutic target of antibacterial agents) and its binding properties were compared with those of ciprofloxacin. Moreover, a field points map of MBA-dMPP ligand was studied to determine electrostatic and van der Waals forces, hydrophobic potentials, and positions involved in ligand-receptor interactions. Finally, the torsion energies of crystal structure and optimized and bioactive conformers of MBA-dMPP were compared to predict its bioactive conformation.

## Introduction

4-Aminoantipyrine (4-amino-1,5-dimethyl-2-phenylpyrazole-3-one) (4-AA) is an analgesic and is used with benzocaine to treat ear pain and discomfort. 4-AA has been reported to exhibit minimal protein binding, to be rapidly, to be completely absorbed in the gastrointestinal tract, and to be extensively metabolized by cytochrome P450 (Poulsen and Loft, 1988[20]). 4-AA is also an intermediate for the preparation of pharmaceuticals, especially antipyretics and analgesics, and for the biochemical production of peroxides or phenols and colorimetric determinations of phenols (Venkateswarlu and Seshaiah, 1995[27]). 4-AA possesses a pyrazolone ring and a free amino group, and therefore, is capable of forming Schiff bases with aldehydes and ketones. To date, a number of Schiff base analogs of 4-AA and their crystal structures have been reported (Li and Zhang, 2006[15]; Hu, 2006[11]; Zhang et al., 2006[30]; Liu et al., 2006[16]; Alam and Lee, 2012[3]; Li et al., 2013[14]). Furthermore, several synthesized Schiff base analogues of 4-AA have been reported to have antibacterial (Alam et al., 2014[4]), antioxidant (Alam and Lee, 2012[3]), cytotoxic (Bensaber et al., 2014[7]), and anti-inflammatory (Alam et al., 2012[2]) activities. Furthermore, transition metal complexes of Schiff base analogues of 4-AA are extensively used in spectrophotometry, chromatography, and electrophoresis, and these complexes have anti-inflammatory, analgesic, antiviral, antipyretic, antirheumatic, and antimicrobial activities.

The crystal structures of biologically active compounds are of great importance for rational drug design. The solid state behaviors of molecules are largely governed by weak interactions, such as, hydrogen bonding and van der Waals interactions, and the prediction of these behaviors is an important goal of supramolecular chemistry (Schmidt et al., 2014[21]; Hu et al., 2014[12]; Tanaka et al., 2014[25]). Hirshfeld surface analysis (Spackman and Jayatilaka, 2009[24]) is an important tool for studying the solid state behaviors of molecules, and allows the different interactions in crystals to be visualized. The structural features of supramolecular systems can be explained using synthons, and combinations of different synthons in supramolecular architectures play significant roles in crystal engineering. Furthermore, study of these intermolecular interactions aids the design of novel compounds with specific structural design requirements and improved properties, which is the main objective of crystal engineering. Furthermore, crystal engineering can be used to optimize the physicochemical properties of Active Pharmaceutical Ingredients (API) in the solid state. Here, we report the synthesis and describe the crystal structure, including the findings of qualitative and quantitative studies of molecular interactions and crystal packing behavior as determined by Hirshfeld surface analysis, of a novel Schiff base derivative of 4-AA, namely, (*E*)-4-(2-methoxybenzylideneamino)-1,5-dimethyl-2-phenyl-1*H*-pyrazol-3(2*H*)-one (MBA-dMPP). Electrostatic potential and geometrical descriptors were analyzed to explain the interactive nature of the surface of MBA-dMPP. In addition, we describe the *in vitro* antibacterial activities of MBA-dMPP against three Gram-positive *(Bacillus cereus*, *Staphylococcus aureus*, and* Listeria* *monocytogenes*), and three Gram-negative bacterial strains (*Salmonella* *tythimurium*, *E*. *coli*, and *Klebsilla* *pneumonia*). *In silico* screening was performed by docking MBA-dMPP using the X-ray crystallographic structure of S12 bacterial protein and filed points analysis was utilized to understand ligand-receptor interactions. Finally, the torsion energies of crystal structure and optimized and docked bioactive conformers of MBA-dMPP were calculated and compared using conformational analyses.

## Material and Methods

### General

The melting point of MBA-dMPP was determined using a Stuart SMP30 melting point apparatus. FT-IR and ^1^H-NMR spectra were recorded on Bruker Tensor 37 (KBr disc) and Bruker 400 MHz (TMS as an internal standard) spectrophotometer, respectively. EI-MS data was obtained using a Jeol JMS700 high-resolution mass spectrometer at the Korea Basic Science Institute (Daegu, Republic of Korea).

### Preparation of (E)-4-(2-Methoxybenzylideneamino)-1,5-dimethyl-2-phenyl-1H-pyrazol-3(2H)-one (MBA-dMPP)

**Figure 1:**
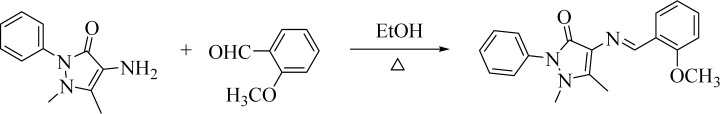
Synthesis of (*E*)-4-[2-methoxybenzylidene amino]-1,5-dimethyl-2-phenyl-1*H*-pyrazol-3(2*H*)-one (MBA-dMPP)

MBA-dMPP was prepared by Aldol condensation as follows: 4-Amino-1,5-dimethyl-2-phenylpyrazol-3-one (203 mg, 1 mmol) was dissolved in anhydrous ethanol (10 mL) and then added to an anhydrous ethanol solution (10 mL) of 2-methoxybenzaldehyde (136 mg, 1 mmol) and refluxed at 80 °C for 3-4h under atmospheric conditions (Figure 1(Fig. 1)). Reaction progress was monitored by TLC. After reaction completion, precipitates were purified by recrystallization from ethanol to provide pure MBA-dMPP. Yield: 87 %, m.p. 212.5-213 °C (yellow crystal). IR (cm^-1^): 3102, 3068, 3009, 2961, 2930, 2838, 1650 (CO), 1592, 1483, 1462, 1436, 1413, 1380, 1362, 1304, 1245, 1199, 1179, 1137, 1105. ^1^H NMR (CDCl_3_, ppm): *δ *2.63 (s, 3H, =C-CH_3_), 3.34 (s, 3H, -N-CH_3_), 3.53 (s, 3H, -OCH_3_), 7.16-7.28 (m, 2H, Ar-H), 7.51-7.75 (m, 7H, Ar-H), 8.18-8.23 (m, 1H, Ar-H), 10.07 (s, 1H, -N=CH). EI-MS m/z ( %): 321 (M^+^, 100), 229 (61), 188(33), 121 (37), 77 (11), 56 (92).

### Crystal structure determination

Yellow needle-shaped crystals of MBA-dMPP were grown by slow evaporation from ethanol solution and a 0.21⨯0.13⨯0.04 mm sized crystal was selected for the X-ray diffraction study. XRD reflection data were collected using a Bruker *SMART* CCD detector and λ (Mo*Kα*) = 0.71073 Å (Bruker AXS Inc., 2000[9]). Of the 70597 reflections collected, 4196 (-9<=h<=9, -18<=k<=18, -23<=l<=23) were treated as observed. Direct methods in SHELXTL NT Version 6.12 (Sheldrick, 2008[23]) were used to determine the coordinates of non-hydrogen atoms. Displacement parameters (isotropic) of C, N, and O atoms were converged to a residual R_1_ of 0.0808 using F2 full-matrix least-squares refinement in SHELXTL. Further refinements (Bruker AXS Inc., 2000[8]) (anisotropic) for C, N and O atoms were carried out using thermal parameters. Hydrogen atoms were fixed at chemically acceptable positions and allowed to ride on parent atoms at C-H distances of 0.94~0.97Å. The final refinement converged to *R* = 0.0411, *wR* = 0.0786, *w* = 1/[σ^2^(*F_ο_*)^2^ + (0.0323*P*)^2^ + 0.32*P*], where P =(max(*F_ο_*^2^, 0 + 2*F*_c_^2^)/3), σ = 1.037, (Δ /σ)_max_<0.001, (Δ*ρ*)_max_ = 0.133 and (Δ*ρ*)_min_ = -0.138 e.Å^-3^.

### Hirshfeld surface analysis

Hirshfeld surface analysis addresses three-dimensional (3D) solid state interactions. The present study, two-dimensional (2D) fingerprint plots of MBA-dMPP were studied using Crystal Explorer 3.1 software (Wolff et al., 2012[28]). Solid state interactions were calculated using:


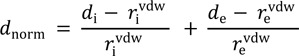


where, *d*_norm _is normalized contact distance, *r*^vdw^ is van der Waals radius, *d*_e_ is distance from the point of interest to the nearest nucleus external to surfaces, and *d*_i _is the distance from a point of interest to the nearest nucleus internal to the surface. A molecular electrostatic potential (MEP) map on the Hirshfeld surface of MBA-dMPP was calculated using Tonto (Jayatilaka and Grimwood, 2003[13]) in Crystal Explorer 3.1, which implements DFT-B3LYP methodology using the standard 3-21G basis set. Geometrical descriptors were calculated to determine surface properties around the molecule using ChemAxon platforms.

### Antibacterial screening

The *in vitro* antibacterial activities of MBA-dMPP were measured against six bacterial strains using the filter paper disc diffusion method (Alam et al., 2011[5]). Briefly, tryptic soya agar (TSA) media (Sigma-Aldrich, MO, USA) was used as the basal medium and media were inoculated with 0.2 mL of 24-h liquid cultures containing the microorganisms. Agar plates (pre-inoculated) containing sample discs were incubated aerobically at 37^ °^C for 24 h. DMSO and ciprofloxacin were used as negative and positive controls, respectively. The diameters of inhibitory zones (in mm) were used to assess inhibitory activities. The minimal inhibitory concentration (MIC, in μg mL^-1^) of MBA-dMPP was determined against *Bacillus cereus* (G^+^), and *Salmonella* *tythi *(G^-^) using nutrient broth medium (DIFCO, Leeuwarden, Netherlands) and a serial dilution technique (Nishina et al., 1987[19]). MIC was defined as the lowest concentration of the tested compound (in DMSO) that inhibited bacterial growth.

### Docking studies

The molecular geometry of MBA-dMPP was investigated using standard bond lengths and angles using the ChemBio3D Ultra 14.0 molecular modeling program (CambridgeSoft Corporation, Cambridge, MA, USA). Molecular energies were minimized using DFT-B3LYP calculations at the 6-311G basis set level in the GAMESS interface of ChemBio3D Ultra 14.0 (PerkinElmer, MA, USA). The crystal structure S12 protein of *E. coli* used for docking studies was obtained from the Protein Data Bank (PDB code: 1FJG). Prior to docking, co-crystallized ligand and water molecules were removed and polar hydrogen atoms and Kollman-united charges were added. AutoDock 4.2 software (Morris et al., 2009[18]) was used to prepare ligand and receptor pdb and pdbqt files. Free rotation was allowed about single bonds during docking. The standard docking protocol was implemented using AutoDock Vina in PyRx 0.8 software (Trott and Olson, 2010[26]) and results were analyzed using Discovery Studio 4.0 (Accelrys, San Diego, CA, USA).

## Results and Discussion

### Synthesis and crystal structure

MBA-dMPP was synthesized (yield 87 %) by condensation between 4-AA (4-amino-1,5-dimethyl-2-phenylpyrazole-3-one; 4-aminoantipyrine) and 2-methoxybenzaldehyde (Figure 1(Fig. 1)).

Purified MBA-dMPP was characterized by FT-IR, ^1^H-NMR, and EI-MS. In its FT-IR spectrum, characteristic bands for carbonyl (C=O) and azomethine (CH=N) were observed at 1650 and 1592 cm^-1^, respectively, and characteristic aromatic and aliphatic C-H stretch absorption bands were evident at 3102-2838 cm^−1^. In its ^1^H NMR spectrum, the characteristic sharp singlet of the azomethine (-CH=N-) proton was observed at δ 10.07 ppm and two singlets of the two -CH_3_ groups attached to the carbon atom and nitrogen of the pyrazolone ring were present at 2.63 and 3.34 ppm, respectively. Methoxy (-OCH_3_) protons generated a singlet at 3.53 ppm, and as was expected, aromatic protons were observed as multiplets at 7.16-7.28 (2H), 7.51-7.75 (7H), and 8.18-8.23 (1H) ppm. In the EI mass spectrum, MBA-dMPP exhibited a molecular ion peak [M]^+^ at m/z 321 at an intensity of 100 %.

The molecular geometry of MBA-dMPP was confirmed by X-ray diffraction using a single crystal obtained by slow evaporation. X-ray diffraction and refinement data are presented in Table 1(Tab. 1).

**Figure 2:**
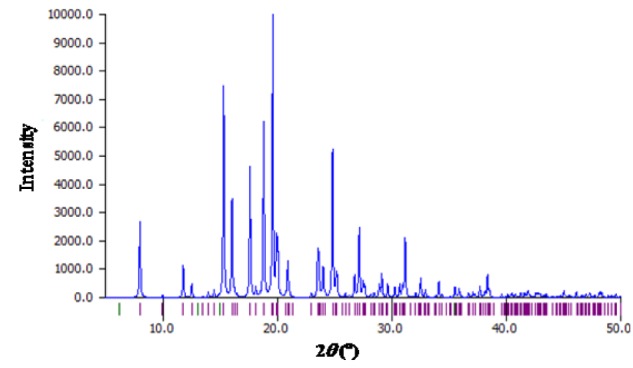
Powder X-ray diffraction pattern of MBA-dMPP after final refinement.

MBA-dMPP crystallized in the orthorhombic system, was of the P2(1)2(1)2(1) space group, and had unit cell parameters of; a = 6.7783(3), b = 14.1162(7), c = 17.6883(10) Å, α= β = γ= 90°, V = 1692.48(15) Å^3^, and Z = 4. Atom coordinates, thermal parameters, and torsion angles are provided in Supporting Information. Selected bond lengths and angles are presented in Tables 2(Tab. 2) and 3(Tab. 3). All bond lengths and angles were within normal ranges. The powder XRD pattern of MBA-dMPP is provided in Figure 2(Fig. 2). The sharpnesses of the peaks obtained indicated good interactions between atoms and x-ray radiation, which is indicative of good crystal quality.

**Figure 3:**
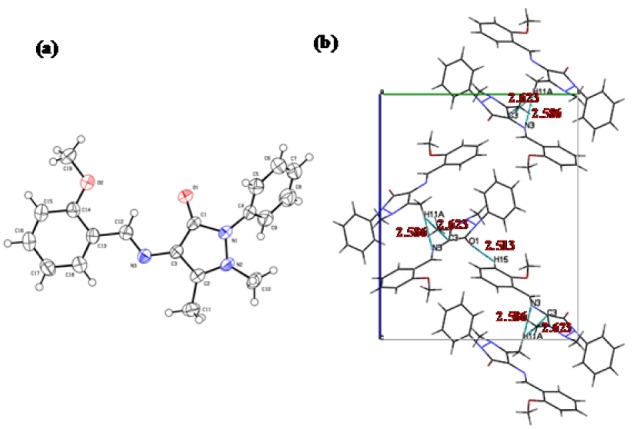
(a) ORTEP drawing of MBA-dMPP and its numbering scheme. Thermal ellipsoids were drawn at the 50 % probability level at 200 K. (b) Packing arrangements in the crystal structure of MBA-dMPP viewed along the “a” axis. Dashed lines indicate close intermolecular contacts.

An ORTEP drawing of the molecular structure of MBA-dMPP (at 50 % probability) and its numbering scheme are provided in Figure 3a(Fig. 3). MBA-dMPP was found to adopt an *E-*configuration about its azomethine group, that is, its -C12=N3- double bond. The bond length of its azomethine group (C12-N3) was 1.286(2)Å and that of its carbonyl (C1-O1) was 1.2308(19)Å; both were within normal reported ranges.

The distance between C2 and C3 in the pyrazolone ring was 1.375(2)Å, indicating a double bond character. In the crystal structure of MBA-dMPP, the five membered antipyrine ring (N1-C1-C3-C2-N2) was almost planar (rmsd 0.0452Å). The dihedral angle between the planes of the antipyrine and phenyl rings (C4-C9) was 69.2(2)° (C1-N1-C4-C5), and the dihedral angles of N2-N1-C4-C5 and N2-N1-C4-C9 were -149.28(16) and 33.2(2)°, respectively. The O1 atom was slightly displaced from the antipyrine mean plane, and the dihedral angles of C2-C3-C1-O1 and N2-N1-C1-O1 were 176.24(18) and -172.24(16)°, respectively. Due to steric hindrance, the N2-methyl and C2-methyl groups were located on opposite sides of the plane of the antipyrine ring and the dihedral angle of C10-N2-C2-C11 was -32.3(3)°. The azomethine double bond (C12-N3) extended conjugation of the double bond of the antipyrine ring (C2-C3) to the benzylidene phenyl ring (C13 to C18), and thus, these two planes were almost coplanar with a dihedral angle of 4.27°. In addition, the plane of the benzylidene phenyl ring (C13 to C18) showed slight deviation from the plane of the azomethine double bond (-C12=N3-) and the dihedral angles of N3-C12-C13-C14 and N3-C12-C13-C18 were -178.90(17) and 0.9(3)°, respectively. The dihedral angles of C18-C13-C14-O2 and C16-C15-C14-O2 were -179.87(16) and 179.6(6)°, respectively. These deviations from planarity were believed to have been caused by intermolecular interactions in the crystal lattice. 

**Figure 4:**
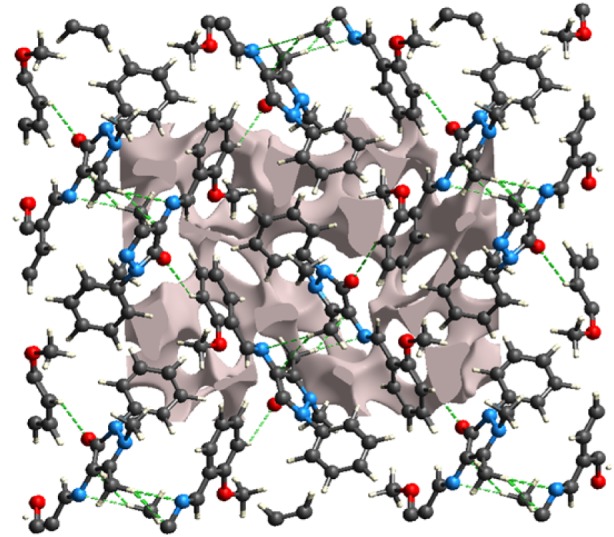
Crystal structure of MBA-dMPP showing voids. *Dashed lines* indicate close intermolecular contacts.

As shown in Figure 3b(Fig. 3), no hydrogen bond was observed in the crystal structure of MBA-dMPP, and thus, structural cohesion was attributed to van der Waals interactions. One unit cell contained four molecules. Two of these molecules were juxtaposed presumably due to edge-edge stacking interactions and each juxtaposed unit was stacked another juxtaposed unit (Figure 3b(Fig. 3)). In particular, N3 and C3 of same molecules were involved in stacking with H11A of an adjacent molecule with distances of 2.586 and 2.623Å, respectively (symmetry operation: -0.5+x, 1.5-y, -z). The O1 of one molecule was involved in edge-edge stacking with H15 of an adjacent molecule; O1 and H15 were separated by 2.513 Å (symmetry operation: 2-x, -0.5+y, 0.5-z). The crystal structure possessed characteristic cavities or voids that could accommodate disordered or diffused solvent molecules. Figure 4(Fig. 4) shows crystal voids of overall volume 222.44 Å^3^, area 708.27 Å^2^, globularity 0.251, and asphericity 0.162, and the total electron count per unit cell (680).

### Molecular interactions

**Figure 5:**
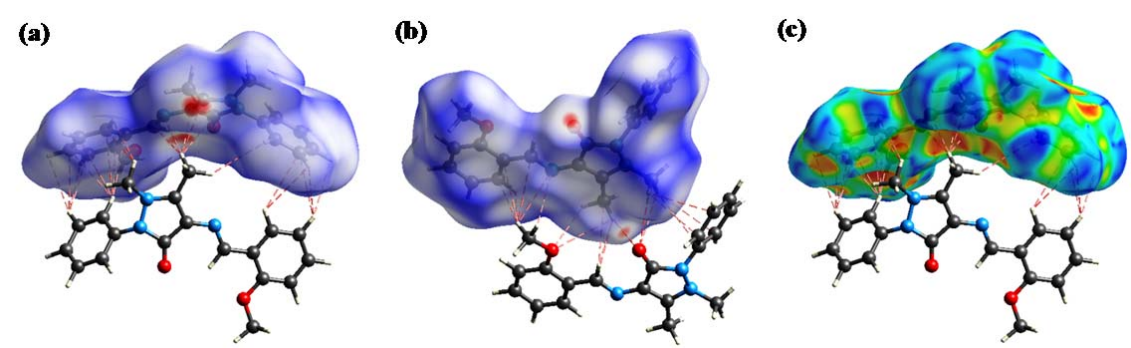
3D Hirshfeld surface *d*_norm_ maps of MBA-dMPP (a: face view and b: rear view). Red rings showing hydrogen bond contacts, and (c) shape index, where red (δ^-^) and blue (δ^+^) surfaces indicate π-π stacking.

Hirshfeld surface analysis (Spackman and Jayatilaka, 2009[24]; Martin et al., 2015[17]) provides a convenient means of studying different types of intermolecular interactions in crystals, because it enables these interactions to be interpreted by visualization. The molecular Hirshfeld surface of MBA-dMPP was generated using CrystalExplorer 3.1 software, and is presented in Figure 5(Fig. 5).

Three-dimensional (3D) Hirshfeld surface maps were obtained using red-white-blue *d*_norm_ surface maps (surface resolution -0.5 to 1.5 Å), where red indicates shorter contacts with negative *d*_norm_ values, white indicates close van der Waals contacts with zero *d*_norm_ values, and blue indicates longer contacts with positive *d*_norm_ values. Strong O…H, N…H and C…H interactions in the crystal structure of MBD-dMPP are shown as deep red areas in Hirshfeld surfaces. Shape index provides a measure of the “shapes” of molecules in lattices, enables complementarity between molecules to be identified, and provides π-π interaction information. Some significant π-π interactions were observed for MBA-dMPP (Figure 5c(Fig. 5); red and blue triangles represent π-π stacking). 

**Figure 6:**
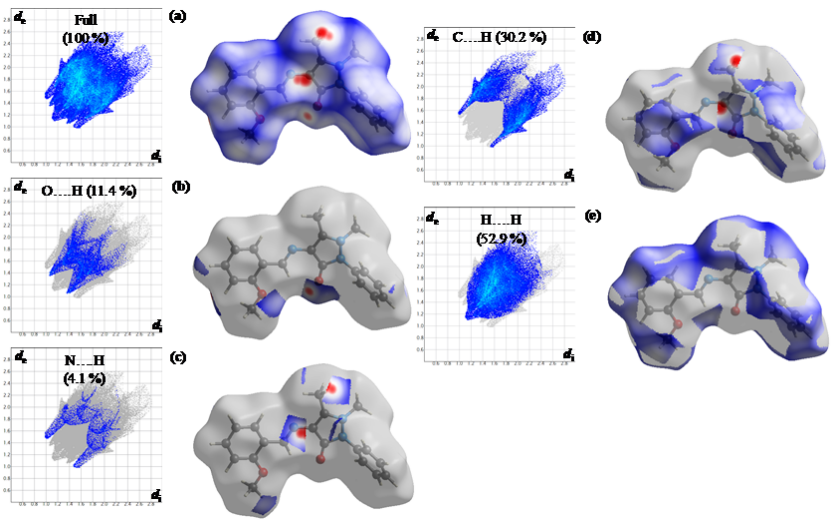
Figure 6: 2D fingerprint maps (left) and corresponding 3D map (right) of molecular interactions of MBA-dMPP, the full map (a) shows reciprocal contacts resolved into: O…H (b), N…H (c), C…H (d) and H…H. (e) shows percentage contact contributions to the total Hirshfeld surface area of the molecule; *d_i_* and *d_e_* are the closest internal and external distances, respectively, from a given point on the Hirshfeld surface.

We also studied 2D fingerprint plots obtained by Hirshfeld surface analysis, which provides information on reciprocal close contacts in crystals. Main reciprocal intermolecular interactions (O…H, N…H, C…H, and H…H) were obtained using a 2D fingerprint plot and 3D *d*_norm_ surfaces of MBA-dMPP (Figure 6(Fig. 6)). Seven spikes were observed in full 2D fingerprint plots (Figure 6a(Fig. 6)) corresponding to O…H, N…H, C…H, and H…H reciprocal close contacts. Figure 6e(Fig. 6) shows H…H interactions contributed most (52.9 %) to the total Hirshfeld surface, as indicated by the middle spike in the 2D fingerprint plot and the blue colored surface in the 3D d_norm _promolecular map. 

These contacts are mainly due to methyl hydrogens of the methoxy and N-methyl groups and to the hydrogen of the aromatic ring. Reciprocal C…H interactions contributed second most (30.2 %) to the total Hirshfeld surface, and are indicated in the 2D finger print plot as spikes on the top left and bottom right and in the 3D d_norm_ surface as red circles (Figure 6d(Fig. 6)). O…H/ H…O (Figure 6b(Fig. 6)) and N…H/H…N (Figure 6c(Fig. 6)) intermolecular contacts appeared as two spikes in 2D fingerprint plots and as red circles in the 3D d_norm_ promolecular surface, which contributed 11.4 % and 4.1 % to the total Hirshfeld surface, respectively. Visible complementary regions in fingerprint plots showed one molecule acts as donor (d_e_>d_i_) and the other as acceptor (d_e _< d_i_). 

**Figure 7:**
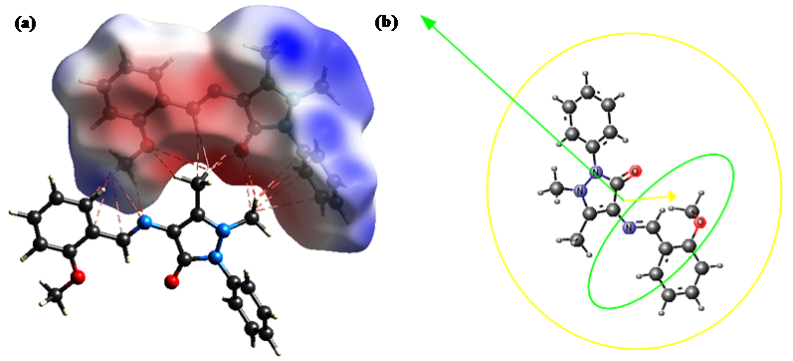
(a) Electrostatic potential mapped on the Hirshfeld surface of MBA-dMPP, where blue and red regions indicating positive and negative potentials, respectively. (b) Geometrical structure of MBA-dMPP showing van der Waals volume and length perpendicular to areas of maximum and minimum potential.

To interpret electrostatic complementarities associated with crystal packing, mean electrostatic potential was mapped on the Hirshfeld surface of MBA-dMPP over the range -0.056 to 0.0.056 au. In Figure 7a(Fig. 7) blue regions correspond to positive electrostatic potentials, indicating hydrogen donor areas in the Hirshfeld surface, and the red regions correspond to negative electrostatic potentials, indicating hydrogen acceptor areas.

The results shown in Figure 7(Fig. 7) were as expected, that is, the positive potential areas of one molecule interact with negative potential areas of another molecule. Geometrical descriptors were also analyzed to provide more information regarding the surface interactions of MBA-dMPP; results are presented in Figure 7b(Fig. 7). The van der Waals volume of MBA-dMPP was 293.22Å^3 ^and Dreiding and MMFF94 energies were 83.18 and 205.91 kcal mol^-1^, respectively. Maximal and minimal projection areas were 100.57 and 44.88 Å^2 ^and maximal and minimal projection radii were 8.21 and 5.10 Å, respectively. Lengths perpendicular to maximum and minimum areas were 7.18 and 15.50 Å, respectively.

### Antibacterial activities

MBA-dMPP was evaluated for *in vitro *antibacterial activity against three Gram-positive bacteria, that is, *Bacillus cereus*, *Staphylococcus aureus*, and *Listeria* *monocytogenes*, and three Gram-negative bacteria e.g. *Salmonella* *tythi*, *E*. *coli*, and *Klebsilla* *pneumonia* by disc diffusion. MBA-dMPP remarkably inhibited the growths of *B. cereus*, *S.* *tythimurium*, *E*. *coli*, and *K.* *pneumonia*, but showed lower activities than ciprofloxacin (positive control) (Table 4(Tab. 4)). Notably, MBA-dMPP exhibited bactericidal activity against all three Gram-negative strains. Minimal inhibitory concentrations (MICs) were also determined against Gram-positive *B. cereus* and Gram-negative *S.* *tythimurium* and MIC values were 12.5 and 50 g mL^-1^, respectively, while ciprofloxacin had MIC values of 6.25 and 1.56 g mL^-1^, respectively.

### Molecular docking and field points studies

**Figure 8:**
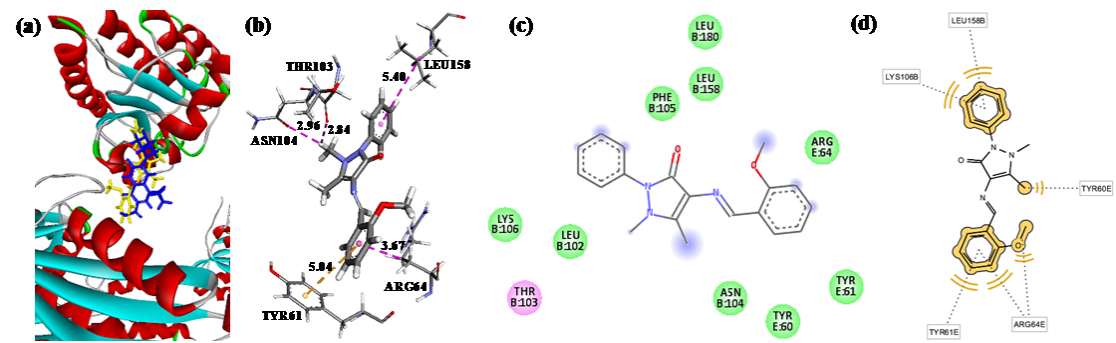
(a) MBA-dMPP (yellow) and ciprofloxacin (blue color) docked in the active site of *E.coli* S12 protein. (b) Binding model of MBA-dMPP with S12 protein (Protein Data Bank entry: 1FJG). The *pink dotted lines* show π-alkyl interactions, and the *yellow dotted lines* show π-pi interactions. (c) 2D ligand interaction diagram with S12 protein obtained using the Discovery Studio program; essential amino acid residues at the binding site are included as tagged circles. *Purple circles* show amino acids that participate in electrostatic and covalent interactions, and the *green circles* show amino acids involved in van der Waals interactions. (d) Pharmacophore model of protein-ligand interactions.

Bacterial S12 protein is promising antibiotic target (Carter et al., 2000[10]), and thus, an useful tool for the *in silico* screening of novel, selective, nontoxic, broad-spectrum antimicrobial drugs. For example, *E*. *coli* S12 was used for *in silico* screening to predict the binding modes of heterocyclics, such as, pyrazolone and oxazolones, and to compare their binding properties with ciprofloxacin (positive control) (Shamsuzzaman et al., 2014[22]; Ahmad et al., 2013[1]). Accordingly, to predict the binding mode of MBA-dMPP, *in silico* docking studies were conducted on its binding to the ciprofloxacin binding site. MBA-dMPP or ciprofloxacin were docked into the active site of *E*. *coli* S12 (PDB ID: 1FJG) using AutoDock 1.5.6 (Morris et al., 2009[18]), AutoDock Vina in PyRx 0.8 software (Trott and Olson, 2010[26]) and binding scores were calculated using iGEMDOCK software (Yang and Chen, 2004[29]). A summary of docking results is presented in Table 5(Tab. 5), and binding models and the different types of interactions found for MBA-dMPP are shown in Figure 8(Fig. 8). The binding site of MBA-dMPP was found to be close to the ciprofloxacin binding site with a RMSD of 1.19 Å ( Figure 8a(Fig. 8)). Docking results showed ciprofloxacin binds with greater affinity to S12 due greater numbers of H-bonds and electrostatic interactions than MBA-dMPP (Table 5(Tab. 5)) and resulted in variation of antibacterial activities between these two compounds. 

**Figure 9:**
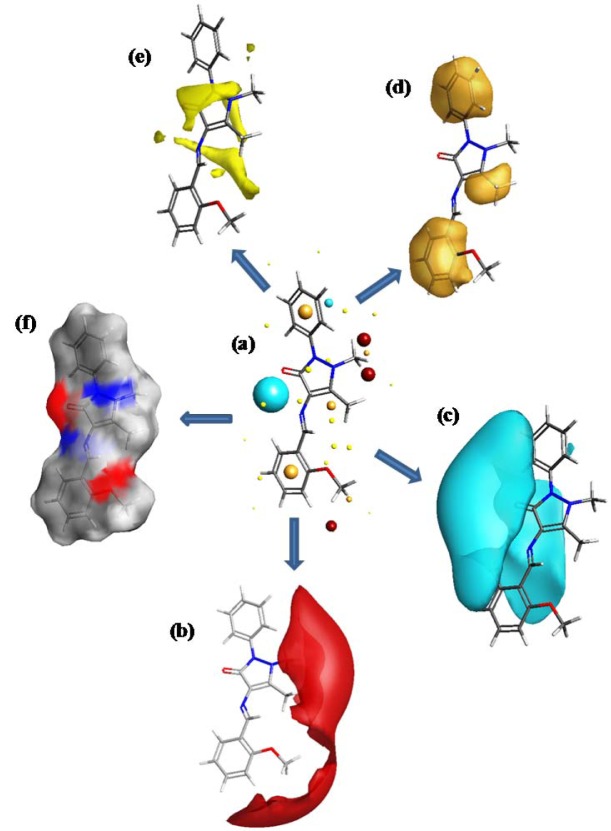
Field patterns and physicochemical properties of MBA-dMPP. (a) The cyan, red, yellow, and gold points show the negative, positive, surface, and hydrophobic fields, respectively, potentially involved in ligand-receptor interactions. (b) Positive field (red) points are presumed to interact with negative/H-bond acceptors on receptors, whereas (c) negative field (cyan) points presumably interact with positive/H-bond donors on receptors. (d) Hydrophobic field (gold) points indicate regions of high polarizability/hydrophobicity. (e) van der Waals surface field (yellow) points are involved in vdW interactions. (f) White smoky regions indicate solvent-accessible surfaces.

The field points (FP) of a molecule provide information about pharmacophores and are used to express ligand-receptor interactions. FP maps consist of electrostatic, van der Waals, and hydrophobic potentials and the locations of interactions. In the present study, we used TorchLite software to study the molecular field point pattern of MBA-dMPP. The molecular field patterns of MBA-dMPP, that is, its electrostatic (positive and negative), steric, and hydrophobic characteristics are presented in Figure 9(Fig. 9). 

The sizes of field points provide information on bonding types and abilities of ligands to interact with receptors - larger field points indicate stronger interactions. Our analysis showed the methyl group attached to the carbon atom of pyrazolone ring and both phenyl rings of MBA-dMPP favored hydrophobic interactions, which concurs with our docking results.

### Conformational analysis

**Figure 10:**
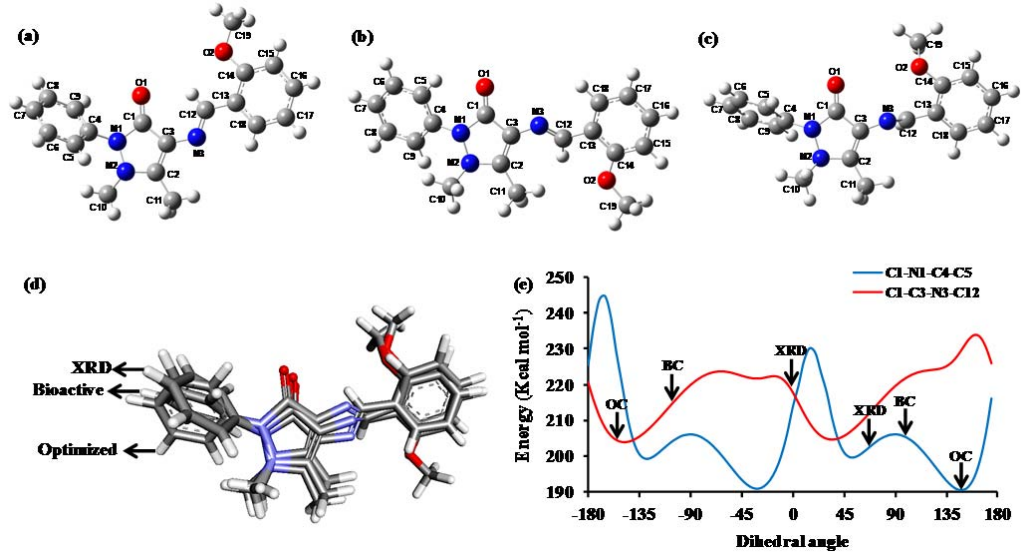
3D geometries of the (a) crystal structure (XRD), (b) optimized conformation (OC) and (c) bioactive conformation (BC) of MBA-dMPP. (d) Superimposition of the optimized conformation and the docked conformation (BC) on the crystal structure (XRD) of MBA-dMPP. (e) Conformational energy curves with dihedral angles (°) between C1-N1-C4-C5 (blue) and C1-C3-N3-C12 (red) as calculated at the B3LYP/6-311G level.

Typically, to interact with receptor sites, bioactive molecules can adopt several preferred conformations of near equal torsion energy by rotating about single bonds. Therefore, to identify the bioactive conformation of MBA-dMPP, we compared by superimposition, its lowest energy optimized conformation (OC) and its docked bioactive conformation (BC), and in addition analyzed its torsion energies, which are related to torsion angles ( Figure 10(Fig. 10)) and are important cheminformatic parameters (Bai et al., 2010[6]). Putative optimized and bioactive conformers were obtained using the DFT-B3LYP/6-311G method and docking calculations, respectively (free rotation was assumed about single bonds). Superimposition ( Figure 10d(Fig. 10)) of these conformations indicated that the dihedral angle between C1-N1-C4-C5 and C1-C3-N3-C12 were significantly different. However, the methoxy group (-OCH_3_) in the benzylidene ring of the OC was displaced by almost 180° from those of the XRD or BC conformers. 

Torsion angles around the C1-N1-C4-C5 bond in XRD, OC, and BC conformers were 69.22, 147.94, and 100.29°, respectively, and corresponding torsion angles around the C1-C3-N3-C12 bond were -4.17, -154.08 and 107.59°, respectively. Therefore, to investigate the conformational energy changes associated with different C1-N1-C4-C5 and C1-C3-N3-C12 torsion angles, a potential energy surface (PES) scan was carried out at the B3LYP/6-311G level by varying torsion angles in 5° increments from -180° to 180° of rotation around N1-C4 and C3-N3 bonds. Analyses of PES scan results showed the torsion energy of the BC conformer (205.36 kcal mol^-1^) around C1-N1-C4-C5 bonds was close to that of the XRD conformer (203.25 kcal mol^-1^), but that the torsion energy of the OC conformer (190.06 kcal mol^-1^) was substantially lower. Similarly, the torsion energies of BC and XRD conformers (214.03 and 220.01 kcal mol^-1^, respectively) around C1-N1-C4-C5 bonds were found to be closer that to that of the OC conformer (204.60 kcal mol^-1^) (Figures **10**a-**10**c(Fig. 10)). Torsion energy analysis and conformer superimposition ( Figure 10d(Fig. 10)) showed that the BC conformer was closes to the XRD conformer than to the OC conformer both energetically and conformationally ( Figure 10e(Fig. 10)).

## Conclusion

We synthesized a novel Schiff base derivative of 4-aminoantipyrine, (*E*)-4-[2-methoxybenzylideneamino]-1,5-dimethyl-2-phenyl-1*H*-pyrazol-3(2*H*)-one (MBA-dMPP), and characterized it by FT-IR, ^1^H-NMR, and EI-MS. The geometry of MBA-dMPP was unambiguously determined by single crystal X-ray diffraction, which revealed it has an orthorhombic (P2(1)2(1)2(1)) symmetry and a *trans* configuration around its central C=N double bond. The unit cell of MBA-dMPP was found to be composed of two pairs of two juxtaposed molecules. The molecule possesses two different planes, that is, the pyrazolone and benzylidene groups are almost coplanar and the phenyl group connected to the N1 atom of the pyrazolone ring constitutes the other plane. Hirshfeld analyses revealed close O…H, N…H, C…H, and H…H contacts and π-π stacking interactions. The H…H and C…H reciprocal contacts contributed 83.1 % to the Hirshfeld surface and 15.5 % of the surface was attributed to O…H/H…O and N…H/H…N interactions. Electrostatic potentials were mapped over the Hirshfeld surface to analyze electrostatic complementarities within the crystal, and geometrical descriptors were also analyzed determine the nature/extent of its surface interactions. The *in vitro* bactericidal activity of MBA-dMPP were examined using three Gram-positive and three Gram negative bacterial strains, and it was found to show highest activity against *B. cereus *and *S. tythimurium* with MIC values of 12.5 and 50 μg mL^-1^, respectively. *In silico* molecular docking studies were performed to investigate interactions between MBA-dMPP and the active site of S12 bacterial protein receptor and these were compared with those of ciprofloxacin. MBA-dMPP was found to bind effectively to the active site of S12 with a high docking score. Finally, electrostatic, van der Waals, hydrophobic potentials, and positions of MBA-dMPP were analyzed further investigate ligand-receptor interactions. Conformation analysis of MBA-dMPP revealed that energetically and conformationally the docked bioactive conformation approximates to its crystal conformation rather than to its optimized conformation. We believe our findings will be found useful by those interested in the design and synthesis of novel antibacterial pyrazolone analogues.

## Supplementary Information

The crystallographic data described in this article has been deposited at the Cambridge Crystallographic Data Center (Deposition number CCDC-1411163). Data can be obtained, free of charge, via http://www.ccdc.cam.ac.uk/conts/retrieving.html, or from the Cambridge Crystallographic Data Centre, 12 Union Road, Cambridge CB2 1EZ, UK; fax: (44) 1223-336033; or e-mail: deposit@ccdc.cam.ac.uk.

## Figures and Tables

**Table 1:**
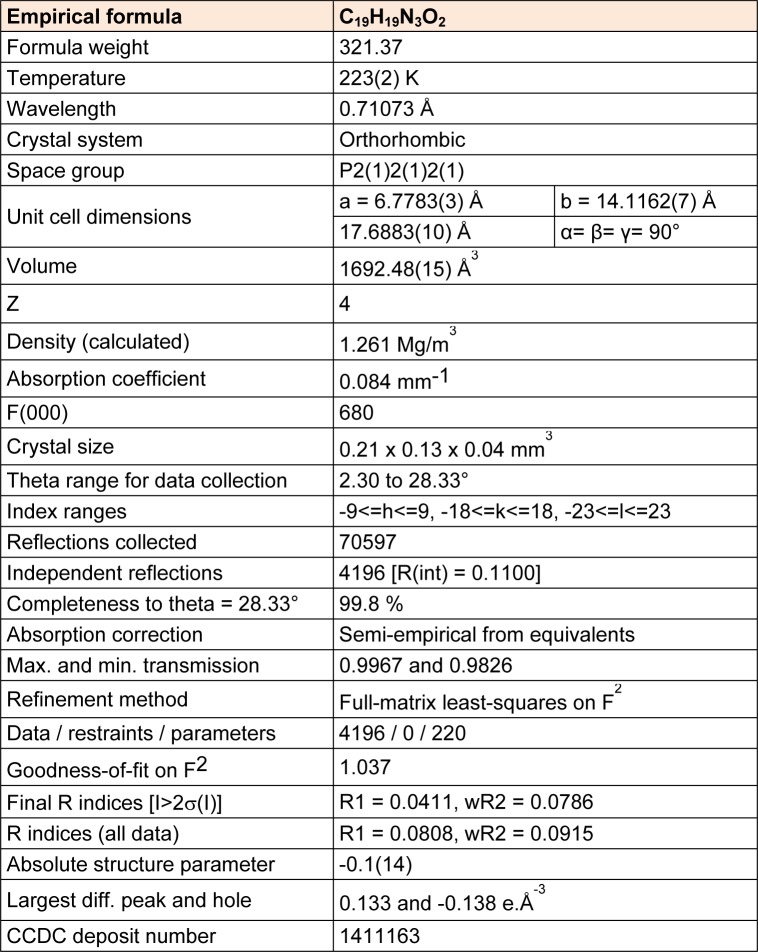
Crystal data, summary of x-ray diffraction intensity and structure refinement data for MBA-dMPP

**Table 2:**
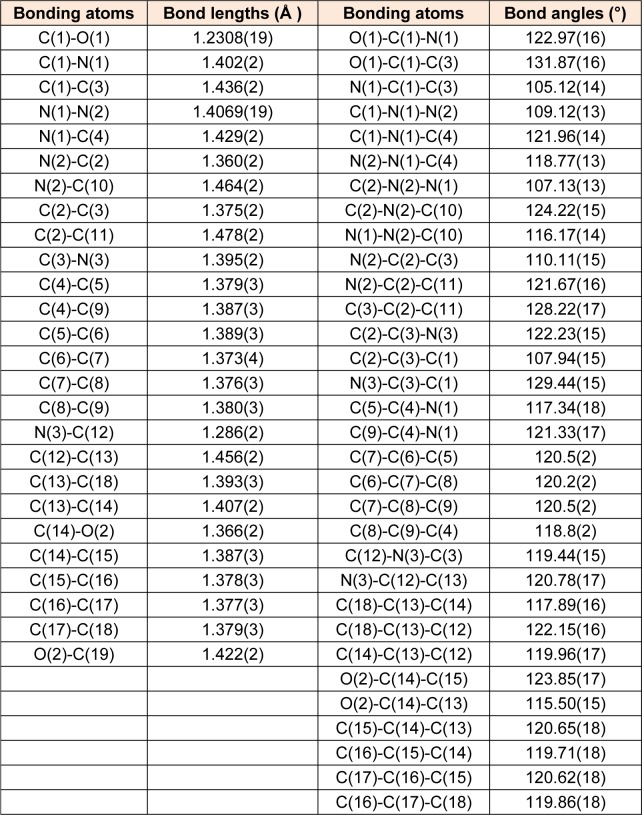
Selected interatomic distances (Å) and valence angles (°) of MBA-dMPP as determined by X-ray crystallography

**Table 3:**
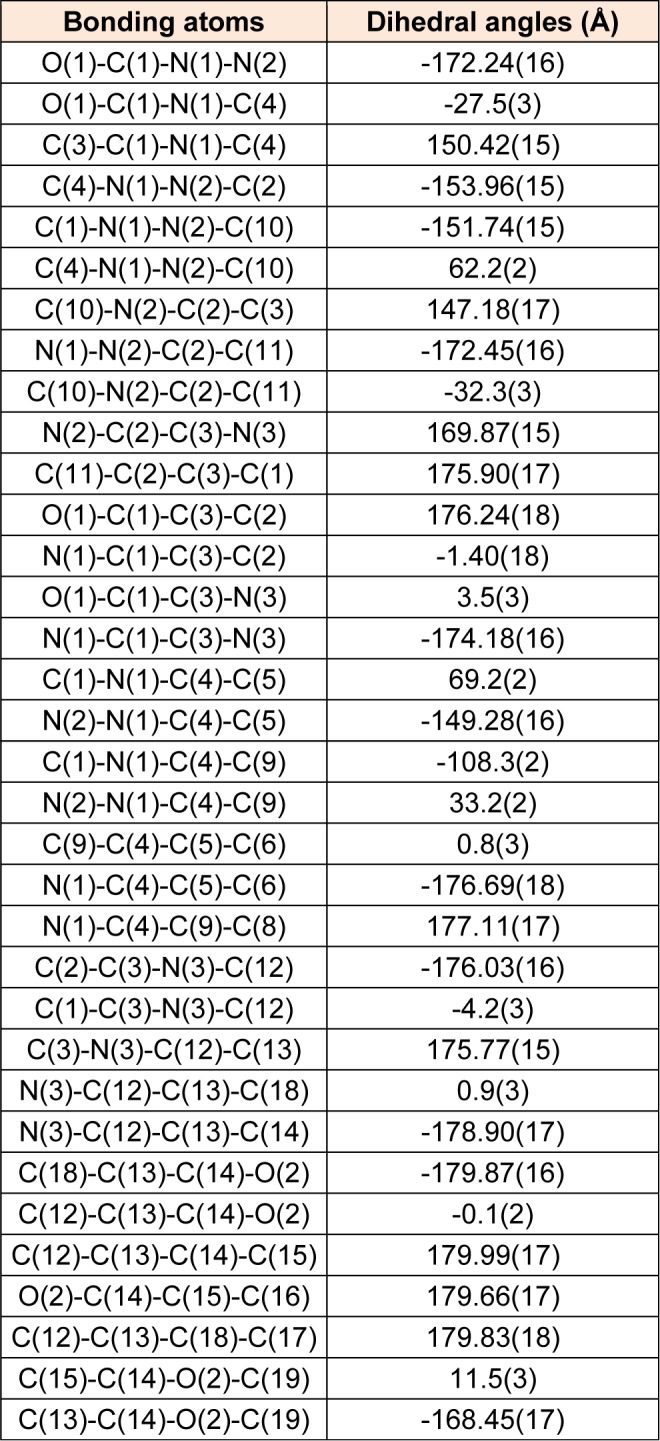
Selected dihedral angles (°) of MBA-dMPP, as determined by X-ray crystallography

**Table 4:**
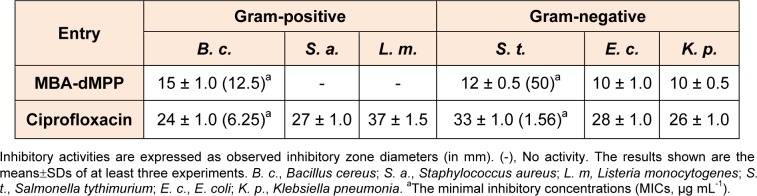
*In* *vitro* bactericidal profiles of MBA-dMPP as determined by measuring zones of inhibition.

**Table 5:**
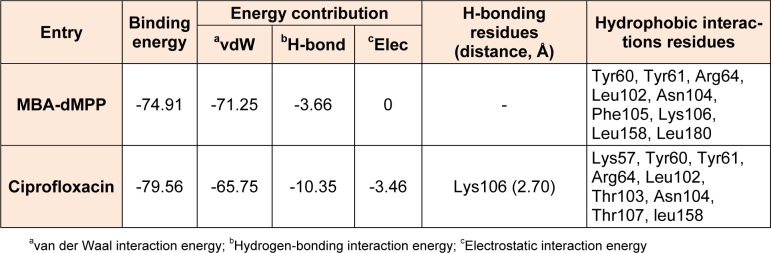
Docking energies and molecular interactions between MBA-dMPP and ciprofloxacin and *E. coli* S12 protein
